# Comparative Outcomes and Efficacy of Programmable Versus Nonprogrammable Ventriculoperitoneal Shunts in the Management of Normal Pressure Hydrocephalus: A Retrospective Study

**DOI:** 10.1155/nri/8882884

**Published:** 2026-03-02

**Authors:** Sultan Jarrar, Mohammed M. Al Barbarawi, Amer Jaradat, Suleiman S. Daoud, Atef F. Hulliel, Teeba Mubaydeen, Sa’ed Hasan, Hamzeh Moh’d Marzouq Bakhiet, Abdulhakim Aldaoud, Adam Abdallah

**Affiliations:** ^1^ Faculty of Medicine, Department of Neurosurgery, King Abdullah University Hospital, Jordan University of Science and Technology, Irbid, Jordan, just.edu.jo; ^2^ Faculty of Medicine, Jordan University of Science and Technology, Irbid, Jordan, just.edu.jo; ^3^ King Hussein Cancer Center, Amman, Jordan, khcc.jo

**Keywords:** normal pressure hydrocephalus, programmable, retrospective study, ventriculoperitoneal shunts

## Abstract

**Background:**

Normal pressure hydrocephalus (NPH) is a neurological disorder in older adults, characterized by gait disturbance, urinary incontinence, and cognitive impairment, along with ventriculomegaly and normal intracranial pressure. The management of NPH often involves ventriculoperitoneal shunting (VPS), which can be programmable (P‐VPS) or nonprogrammable (NP‐VPS). While P‐VPS offers the advantage of adjustable pressure settings, its impact on clinical outcomes and complications remains debated, particularly in resource‐limited settings like Jordan.

**Method:**

A retrospective review was conducted of 38 adult patients diagnosed with idiopathic NPH who underwent VPS placement between 2018 and 2024. Patients were classified into two groups: P‐VPS and NP‐VPS. Clinical outcomes, including symptom improvement, complication rates, hospital stay duration, and shunt revisions, were analyzed. Statistical comparisons were made using SPSS, with *p* values < 0.05 considered significant.

**Results:**

The study found no significant differences between the two groups in symptom improvement. However, the NP‐VPS group had a significantly shorter hospital stay (5.7 ± 3.2 days vs. 14.1 ± 11.9 days, *p* = 0.007). Complication rates, including infection and shunt revision, were higher in the P‐VPS group (20.0% vs. 7.7% for infection; 32.0% vs. 15.4% for revision), though differences were not statistically significant.

**Conclusion:**

Both P‐VPS and NP‐VPS resulted in similar symptom improvements, with NP‐VPS showing a trend toward shorter hospital stays and comparable complication rates. Further multicenter studies with larger sample sizes are needed to validate these findings and refine management strategies for NPH.

## 1. Introduction

Normal pressure hydrocephalus (NPH) is a chronic progressive disease and represents the majority of hydrocephalus cases in adults. Patients present with a triad of symptoms: compromised gait, cognitive impairment, and urinary incontinence, associated with marked dilation of cerebral ventricles and normal intracranial pressure. Symptoms may manifest asynchronously or incompletely [1, 2]. Notably, NPH is a common neurologic condition in adults, with an estimated global prevalence of 85 per 100,000, and its incidence is projected to increase in parallel with population aging [3]. The symptoms of NPH are often debilitating and can be misdiagnosed as other neurodegenerative conditions, such as Parkinson’s disease or Alzheimer’s disease, underscoring the importance of accurate diagnosis and timely intervention [4].

Despite the evolution of clinical and radiological evaluation of the disease, understanding of the pathogenesis of the disease is limited, and its management remains a challenge.

A reduction in elastic compliance of the cerebral blood vessels and ventricular walls with aging is a plausible pathophysiological mechanism, and ventriculoperitoneal shunt (VPS) still stands as the mainstay treatment for symptomatic relief [5, 6].

However, VPS is not without significant drawbacks. Shunt surgery is associated with a high rate of complications and subsequent shunt revisions. [7, 8], with reported complication rates in adults ranging from 17% to 33% [9, 10]. These complications include fatal infections, dysfunction, obstruction, effusion, and over‐drainage, causing subdural hematoma [11, 12]. The high rate of complications and the need for revision surgery represent a substantial burden on patients and healthcare systems alike.

VPS can further be classified into programmable VPS (P‐VPS) and nonprogrammable VPS (NP‐VPS), according to their ability to regulate the valve opening pressure (OP) post‐VPS implantation (pressure‐regulated), based on the clinical signs of cerebrospinal fluid (CSF) drainage. P‐VPS allows for adjustable pressure settings to optimize CSF drainage [10].

Over the last two decades, the use of P‐VPS has increased, with an increasing need for studies comparing it to N‐VPS in terms of efficacy and safety. Although P‐VPS is reported to potentially improve patient outcomes and reduce complications, its higher cost prompts questions about its worth [13].

In this context, selecting the appropriate valve setting and ensuring early detection of shunt‐related complications are key aspects of NPH management. A paucity of information is available in the literature to make conclusions on the use of either type [14]. In this study, we aim to analyze the clinical outcomes and complications of NPH patients treated with VPS, while comparing P‐VPS to NP‐VPS. Specifically, this study addresses a gap in the literature by retrospectively comparing clinical outcomes, complication rates, and resource utilization, particularly hospital length of stay, in a cohort of NPH patients treated at a tertiary care center in Jordan. As the first study of its kind in Jordan and the region, it seeks to provide valuable insights and guide healthcare institutions toward the adoption of the most optimal VPS approach for managing NPH.

## 2. Materials and Methods

### 2.1. Study Design and Data Collection

We performed a retrospective review of adult patients with an established diagnosis of iNPH who underwent VPS placement between 2018 and 2024. Diagnosis of iNPH was determined based on internationally accepted criteria, which included age over 40 years at symptom onset; the presence of at least one symptom from the classic clinical triad, gait disturbance, urinary incontinence, or cognitive impairment; and neuroimaging evidence of ventriculomegaly disproportionate to cortical atrophy, with or without periventricular signal changes. In cases where available, a normal CSF OP (< 20 cm H_2_O) on lumbar puncture (LP) was required, and a positive clinical response to a high‐volume LP or external lumbar drainage was used to further support the diagnosis. In a few cases, VPS placement was performed despite lack of LP improvement, guided by clinical assessment and supportive cisternography results. Patients who had other diagnoses, were treated outside the hospital, or were lost to follow‐up were excluded. A total of 38 patients fulfilled the inclusion criteria and were included in the final analysis. The initial dataset was obtained through the King Abdullah University Hospital (KAUH) registry and supplemented with variables regarding patient and disease characteristics. Clinical data were extracted from electronic health records, including demographics (age, sex, and BMI), presenting symptoms (gait disturbance, urinary incontinence, and cognitive impairment), and the presence of the complete clinical triad. Preoperative imaging data were reviewed, including the use of CT, MRI, and cisternography, as well as OP and CSF volume when available. Patient response to a diagnostic LP was assessed based on documented symptom improvement in gait, urinary function, or memory. Symptom improvement was categorized into three phases based on the timing and progression of recovery and follow‐up period within one year. The first phase represented the initial symptom to improve, the second phase reflected any subsequent symptom improvements reported in later clinical encounters, and the third phase captured additional recovery beyond the second improvement. The choice between programmable and nonprogrammable VPS valves was not governed by a standardized institutional protocol. Valve selection was determined retrospectively and was primarily based on the operating surgeon’s preference, patient financial considerations or insurance coverage, and the availability of specific valve models at the time of surgery.

### 2.2. Ethical Approval

All subjects participated voluntarily. The study was approved by the institutional review board of KAUH (IRB Ref.: 2024/444). The Declaration of Helsinki was adequately addressed. Informed consent was waived by the institutional review board due to the retrospective nature of the study, under the condition of maintaining data confidentiality and using it only for scientific purposes.

### 2.3. Outcomes and Definitions

Outcomes assessed included symptom improvement, complication rates, shunt revisions, and length of hospital stay. Shunt revision was defined as any return to the operating room for shunt adjustment, replacement, or removal. Symptom improvement following VPS placement was categorized into three phases based on timing and progression during the follow‐up. The first phase represented the initial symptom to improve. The second phase reflected any subsequent symptom improvements observed in later clinical encounters. The third phase captured additional recovery beyond the second improvement.

### 2.4. Statistical Analysis

All data analyses were performed using the IBM Statistical Package for the Social Sciences (SPSS) software for Windows, Version 26.0 [15]. Descriptive measures included mean ± standard deviations for continuous data if the normality assumption was not violated, according to the Shapiro–Wilk test, and median with first and third quartiles (Q1–Q3) if the assumption was violated. Categorical data were presented by frequencies and percentages (%). The chi‐square test was used for categorical variables, and Fisher’s exact test was applied when expected frequencies were below the acceptable threshold.

## 3. Results

### 3.1. Study Population and Clinical Presentation

A total of 38 patients with NPH were included, with 25 receiving programmable shunts and 13 receiving nonprogrammable shunts. The mean age was comparable between groups (66.9 ± 13.2 years vs. 66.6 ± 10.4 years, respectively). The proportion of female patients was higher in the programmable shunt group (40.0%) compared to the nonprogrammable group (7.7%). BMI was similar in both groups (29.6 ± 5.1 vs. 29.8 ± 6.0, respectively). All patients exhibited gait disturbance (100%), while urinary incontinence and dementia were present in 81.6% and 76.3% of the total sample, respectively. The prevalence of these symptoms did not differ significantly between groups (*p* > 0.05 for all symptoms). Additionally, 65.8% of patients presented with the complete triad of symptoms (gait, urinary, and memory symptoms) (Table 1).

**TABLE 1:** Characteristics of included patients.

Variables	*N* (%)		
	Total *N* = 38	Programmable *N* = 25	Non‐programmable *N* = 13
Age			
Mean (SD)	66.8 (12.2)	66.9 (13.2)	66.6 (10.4)
Sex			
Female	11 (28.9)	10 (40.0)	1 (7.7)
Male	27 (71.1)	15 (60.0)	12 (92.3)
BMI			
Mean (SD)	29.6 (5.4)	29.6 (5.1)	29.8 (6.0)
Symptoms			
Gait	38 (100.0)	25 (100.0)	13 (100.0)
Urine	31 (81.6)	21 (84.0)	10 (76.9)
Dementia	29 (76.3)	19 (76.0)	10 (76.9)
Triad	25 (65.8)	18 (72.0)	7 (53.9)

### 3.2. Imaging

Preoperative imaging modalities performed are listed in Table 2. CT scans were obtained in 73.7% and MRI in 84.2% of cases. Cisternography was performed in 55.3% of patients. Among the 19 patients with available cisternography reports, findings were consistent with NPH in 79.0% of cases, with similar proportions in both groups (*p* = 0.603). The mean OP was 13.0 ± 3.7 cm H_2_O, programmable (12.8 ± 4.4 cm H_2_O) and nonprogrammable (13.3 ± 2.2 cm H_2_O) groups. CSF volume also showed no significant differences between groups (35.3 ± 12.4 vs. 37.2 ± 8.0 mL, respectively, *p* = 0.618).

**TABLE 2:** Investigations undertaken by patients and their findings.

Variable	*N* (%)		
	Total *N* = 38	Programmable *N* = 25	Nonprogrammable *N* = 13
Investigations done			
CT	28 (73.7)	19 (76.0)	9 (69.2)
MRI	32 (84.2)	21 (84.0)	11 (84.6)
Cisternography	21 (55.3)	13 (52.0)	8 (61.5)
Cisternography findings^∗^			
Consistent with NPH	15 (79.0)	10 (83.3)	5 (71.4)
Not consistent with NPH	4 (21.0)	2 (16.7)	2 (28.6)
Opening pressure			
Mean (SD)	13.0 (3.7)	12.8 (4.4)	13.3 (2.2)
CSF volume			
Mean (SD)	36.0 (11.0)	35.3 (12.4)	37.2 (8.0)

^∗^ *N* = 19, who underwent cisternography with radiology report available.

### 3.3. Improvement

**FIGURE 1:**
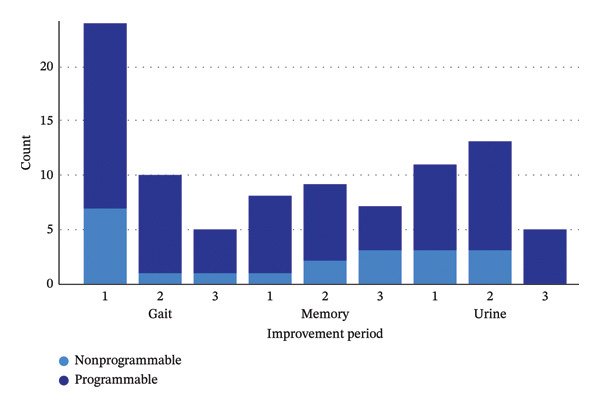
First symptom to improve following VPS placement in NPH patients stratified by the shunt type: programmable VPS and nonprogrammable VPS.

Following LP, 76.3% of patients showed improvement, with no significant difference between the programmable (80.0%) and nonprogrammable (69.2%) shunt groups (*p* = 0.689). Regarding the first symptom to improve, gait disturbance was the most commonly reported improvement (63.2%), followed by urinary symptoms (28.9%) and memory function (21.1%). There were no significant differences between the two groups in terms of first symptom improvement (*p* > 0.05 across all symptoms). In the second phase of symptom improvement, 47.4% of patients reported further recovery, with gait (26.3%), urinary function (34.2%), and memory (23.7%) showing progressive improvements. However, there were no statistically significant differences between the two groups (*p* > 0.05 for each symptom observed). By the third phase of improvement, 28.9% of patients exhibited further symptom recovery. Gait improvement was noted in 13.2%, urinary symptoms in 13.2%, and memory in 18.4%. No significant differences were observed between programmable and nonprogrammable shunt groups in any category (*p* > 0.05) (Figure 1: first symptom to improve following VPS placement in NPH patients stratified by the shunt type: programmable VPS and nonprogrammable VPS). Detailed symptom improvement outcomes by shunt type are present in Table 3.

**TABLE 3:** Symptom improvement outcomes by shunt type.

Variable	*N* (%)			*p* value
	Total *N* = 38	Programmable *N* = 25	Nonprogrammable *N* = 13	
Outcome after LP				
Improvement	29 (76.3)	20 (80.0)	9 (69.2)	
No improvement	9 (23.7)	5 (20.0)	4 (30.8)	
First improvement				
No improvement				
No	29 (76.3)	19 (76.0)	10 (76.9)	
Yes	9 (23.7)	6 (24.0)	3 (23.1)	
Gait				0.486
No	14 (36.8)	8 (32.0)	6 (46.2)	
Yes	24 (63.2)	17 (68.0)	7 (53.8)	
Urine				0.714
No	27 (71.1)	17 (62.0)	10 (76.9)	
Yes	11 (28.9)	8 (32.0)	3 (23.1)	
Memory				0.222
No	30 (78.9)	18 (72.0)	12 (92.3)	
Yes	8 (21.1)	7 (28.0)	1 (7.7)	
Second improvement				
No improvement				
No	18 (47.4)	13 (52.0)	5 (38.5)	
Yes	20 (52.6)	12 (48.0)	8 (61.5)	
Gait				0.118
No	28 (73.7)	16 (64.0)	12 (92.3)	
Yes	10 (26.3)	9 (36.0)	1 (7.7)	
Urine				0.473
No	25 (65.8)	15 (60.0)	10 (76.9)	
Yes	13 (34.2)	10 (40.0)	3 (23.1)	
Memory				0.456
No	29 (76.3)	18 (72.0)	11 (84.6)	
Yes	9 (23.7)	7 (28.0)	2 (15.4)	
Third improvement				
No improvement				
No	11 (28.9)	7 (28.0)	4 (30.8)	
Yes	27 (71.1)	18 (72.0)	9 (69.2)	
Gait				0.643
No	33 (86.8)	21 (84.0)	12 (92.3)	
Yes	5 (13.2)	4 (16.0)	1 (7.7)	
Urine				0.144
No	33 (86.8)	20 (80.0)	13 (100.0)	
Yes	5 (13.2)	5 (20.0)	0 (0.0)	
Memory				0.672
No	31 (81.6)	21 (84.0)	10 (76.9)	
Yes	7 (18.4)	4 (16.0)	3 (23.1)	

### 3.4. Complications

Table 4 presents the complication rates across the two shunt types. The mean duration of stay was significantly shorter in the nonprogrammable shunt group (5.7 ± 3.2 days) compared to the programmable shunt group (14.1 ± 11.9 days; *p* = 0.007). These results should be interpreted with caution due to the small sample size and data variability. While the incidence of infection was higher in the programmable shunt group (20.0%) compared to the nonprogrammable group (7.7%), this difference was not statistically significant (*p* = 0.643). Similarly, the rate of shunt revision was also higher in the programmable shunt group (32.0%) compared to the nonprogrammable group (15.4%), but this difference did not reach statistical significance (*p* = 0.441). These differences are presented as observed trends due to the small sample size.

**TABLE 4:** Complication rate across shunt types.

Variable	*N* (%)			*p* value
	Total *N* = 38	Programmable *N* = 25	Nonprogrammable *N* = 13	
Duration of stay				0.007
Mean (SD)	11.2 (10.5)	14.1 (11.9)	5.7 (3.2)	
Infection				0.643
No	32 (84.2)	20 (80.0)	12 (92.3)	
Yes	6 (15.8)	5 (20.0)	1 (7.7)	
Shunt revision				0.441
No	28 (73.7)	17 (68.0)	11 (84.6)	
Yes	10 (26.3)	8 (32.0)	2 (15.4)	

## 4. Discussion

In this retrospective study conducted at a tertiary center over six years, we compared the clinical outcomes associated with P‐VPS versus NP‐VPS in managing NPH. Our findings contribute to the ongoing discussion regarding the optimal shunt type for NPH, highlighting real‐world considerations in balancing efficacy, safety, and resource utilization, particularly in a resource‐limited setting like Jordan.

The choice between P‐VPS and NP‐VPS remains a subject of debate in neurosurgical practice, particularly concerning long‐term outcomes and cost‐effectiveness [16]. Current international guidelines often favor the use of P‐VPS due to the flexibility they offer in postoperative pressure adjustments, which can mitigate complications such as overdrainage or underdrainage without the need for revision surgery [17, 18].

The demographic characteristics of age and BMI in the 38 patients included in our cohort were comparable between both groups, with a higher proportion of female patients noted in the P‐VPS group (40.0% vs. 7.7%). The mean age of the patients in our cohort was 66.8 ± 12.2 years, aligning with the geriatric population typically affected by idiopathic NPH [19, 20]. Gait disturbance was the most frequently improved symptom (63.2%). This aligns with prior literature, which consistently identified gait disturbance as the most responsive symptom to CSF diversion in NPH patients [21, 22].

P‐VPS and NP‐VPS were associated with similar rates of symptom improvement following LP and shunt placement in our analysis. The lack of significant differences in symptom improvement between groups with both shunt types suggests that the ability to adjust valve settings in P‐VPS may not confer a clear advantage in achieving symptomatic relief in the short term. This finding is consistent with the results reported by Türkkan and Bekar, who also found no significant difference in symptom improvement between programmable and nonprogrammable shunts [23].

In terms of complications, a key finding of this study is the significantly shorter hospital stay in the NP‐VPS group compared to the P‐VPS group (5.7 vs. 14.1 days; *p* = 0.007); however, this finding should be interpreted as an association rather than a causal effect due to the small sample size. This difference may also reflect the need for prolonged monitoring or adjustments in patients with P‐VPS to optimize valve settings postimplantation. While P‐VPS allows for noninvasive pressure adjustments, the process of titrating settings to achieve optimal CSF drainage may extend hospitalization, particularly in complex cases. Given that both shunt types showed comparable clinical efficacy in terms of symptom improvement, the difference in hospital stay represents a key factor in evaluating resource utilization, particularly in a resource‐limited setting. Shorter hospital stays are generally associated with reduced costs and a lower risk of nosocomial complications. While the initial cost of P‐VPS is higher, often reported to be two to three times that of NP‐VPS [16, 24], the total cost of care is influenced not only by device price but also by the duration of hospitalization. Therefore, the longer stay associated with P‐VPS may contribute substantially to healthcare expenditure, suggesting that NP‐VPS could be a more economically favorable option in many cases. Nonetheless, P‐VPS may remain advantageous in selected patients, such as those with fluctuating symptoms or a higher risk of over‐ or underdrainage, where noninvasive valve adjustments could prevent the need for surgical revision.

Complication rates, including infection and shunt revision, were higher in the P‐VPS group (20.0% vs. 7.7% for infection; 32.0% vs. 15.4% for shunt revision), though these differences did not reach statistical significance (*p* = 0.643 and *p* = 0.441, respectively). Regarding infection rates, these findings are consistent with the meta‐analysis by Katiyar et al., which also found similar infection rates between programmable and nonprogrammable shunts, with no statistically significant differences [10]. Regarding shunt revision, while our analysis did not find a statistically significant difference, several studies in the literature have reported a higher revision rate in the NP‐VPS group, although the results were not consistent. Some studies found no statistically significant difference [25], while others observed a significantly higher revision rate in the NP‐VPS group [13, 26].

The trend toward higher complications in the P‐VPS group may be attributed to the complexity of programmable valves, which are more susceptible to mechanical failure or maladjustment.

Additionally, the need for repeated valve adjustments in P‐VPS may increase the risk of infection due to more frequent clinical interventions [27].

It should be noted that, in our study, the criteria for selecting a P‐VPS versus an NP‐VPS were not formalized in a standardized institutional protocol. Valve selection was primarily guided by a combination of factors, including the operating surgeon’s preference, the patient’s financial status or insurance coverage, and the availability of specific shunt models at the time of surgery.

Our study has a few limitations that should be considered. First, the retrospective, nonrandomized design may introduce some level of selection bias. Additionally, differences in shunt selection may be influenced by factors such as patient complexity, surgeon preference, or timing, rather than solely reflecting the performance of the device. To address this in future studies, prospective designs with larger, more diverse patient cohorts could provide more robust evidence. Second, the relatively small sample size (*n* = 38) may have limited the statistical power to detect significant differences in complication rates. Future research with larger sample sizes could better assess the risks associated with NP‐VPS. Finally, the study was conducted at a single center, which may limit the generalizability of the findings. Multicenter, cost‐effective studies involving various healthcare settings and patient populations could help validate these results and improve the applicability of the findings.

## 5. Conclusion

This study compares the clinical outcomes of P‐VPS and NP‐VPS in the management of NPH among patients in Jordan. Both shunt types resulted in similar symptom improvements, while the P‐VPS group showed a trend toward longer hospital stays, potentially related to valve adjustment complexities. Although complication rates appeared higher in the P‐VPS group, these differences were not statistically significant. Given these observations, NP‐VPS may be associated with shorter hospital stays and comparable clinical outcomes; however, these findings should be interpreted cautiously due to the small sample size and single‐center setting. As the first study of its kind in Jordan and the region, our findings are intended to provide initial insights and help guide future research. Prospective, multicenter studies with larger cohorts are warranted to validate these trends and further guide optimal VPS management in NPH.

## Author Contributions

Sultan Jarrar: conceptualization, methodology, project administration, and writing–original draft preparation. Mohammed M. Al Barbarawi: supervision. Amer Jaradat: supervision and writing–review and editing. Suleiman S. Daoud: supervision and writing–review and editing. Atef F. Hulliel: conceptualization, methodology, and writing–original draft preparation. Teeba Mubaydeen: writing–review and editing. Sa’ed Hasan: formal analysis. Hamzeh Moh’d Marzouq Bakhiet: data curation. Adam Abdallah: data curation. Abdulhakim Aldaoud: writing–review and editing.

## Funding

The authors have nothing to report.

## Ethics Statement

All subjects participated voluntarily. The study was approved by the institutional review board of King Abdullah University Hospital (KAUH) (IRB Ref: 2024/444). The Declaration of Helsinki was adequately addressed. Informed consent was waived by the institutional review board due to the retrospective nature of the study.

## Conflicts of Interest

The authors declare no conflicts of interest.

## Data Availability

The data that support the findings of this study are available from the corresponding author upon reasonable request.
